# Relay Selection for Covert Communication with an Active Warden

**DOI:** 10.3390/s25133934

**Published:** 2025-06-24

**Authors:** Jong Yeol Ryu, Jung Hoon Lee

**Affiliations:** 1 Department of AI Information Engineering, Gyeongsang National University, Jinju 52828, Republic of Korea; jongyeol_ryu@gnu.ac.kr; 2 Department of Electronics Engineering and Applied Communications Research Center, Hankuk University of Foreign Studies (HUFS), Yongin 17035, Republic of Korea

**Keywords:** covert communication, active warden, relay selection, power allocation, covert rate maximization

## Abstract

In this paper, we consider covert communication with multiple relays and an active warden who not only sends jamming signals but also aims to detect the covert transmission. In the relay system with the active warden, the most critical factor is the channel between the relay and the warden, as the warden leverages this channel to transmit jamming signals while trying to detect the presence of covert communication. To mitigate the impact of the active warden, we propose a relay selection scheme that selects the relay with the minimum channel gain to the warden. We analyze the performance of the proposed scheme and demonstrate how increasing the number of relays leads to performance improvements based on analytical results. Numerical results show that the analytical predictions closely match the simulations, and our proposed scheme effectively increases the covert rate while minimizing the threat posed by the active warden.

## 1. Introduction

Recently, as the importance of privacy and information security has grown significantly, interest in security technology has also increased substantially. The traditional approach to information security relies on cryptographic techniques at the upper layers of communication systems. However, advances in devices with powerful computational capabilities have placed cryptographic technology under increasing threat. As a result, physical layer security technology was introduced to enhance security across all communication layers [1]. The goal of physical layer security technology is to prevent information eavesdropping by unauthorized eavesdroppers by exploiting the properties of the wireless channel, such as fading and noise. Pioneering researchers have demonstrated that a positive information-theoretic secrecy capacity can be achieved for various channel models [2,3,4]. Building on the knowledge of information-theoretic capacity, efficient transmission strategies have been proposed and analyzed for physical layer security. However, as the demand for strong security on the physical layer has increased, there has been a growing need for more robust security techniques that could conceal the existence of the transmission. To address this demand for stronger security, covert communications have been introduced as a key security technology for future communication networks [5,6] such as industrial Internet of Things (IoT), autonomous robotic systems, and UAV swarms, where communication often takes place in adversarial or sensitive environments. In such scenarios, undetectable data exchange is essential to ensure mission integrity and prevent interception or jamming. The goal of covert communication techniques is to prevent the exposure of the transmission’s presence itself to wardens. As a result, covert communications provide stronger security compared to conventional physical layer security techniques, where messages may be received by eavesdroppers but remain undecodable.

Various communication strategies have been investigated to increase the covert rate for covert communication [7,8,9,10]. Channel inversion power control for covert communication was proposed in [7], and it was shown that a positive covert rate can be achieved with the aid of a full-duplex receiver. A stochastic geometric approach to analyzing covert communication networks was presented in [8]. For a random network where wardens and friendly jammers are located randomly, the authors maximized the covert rate through transmit power and rate control. In [9], an opportunistic jammer selection strategy was proposed for covert communication with multiple friendly jammers. The authors first analyzed the detection error and outage probabilities and then obtained the optimal threshold for jammer selection based on analytical results. For multiple-antenna covert communication systems, efficient beamforming techniques were proposed in [10]. The zero-forcing beamformer and the robust beamformer were designed for perfect channel state information (CSI) and imperfect CSI scenarios, respectively.

As a promising technique to achieve covertness, relay-assisted transmission has also been intensively studied [11,12,13,14]. In covert communication systems, relays can be utilized both as jammers to confuse the warden’s detection and as helpers to forward the covert message from the transmitter. The square root law was demonstrated in relay-assisted covert communications [11]. In [11], it was shown that the square root law can be achieved with the help of a full-duplex amplify-and-forward (AF) relay and channel uncertainty. Covert rate maximization was considered for covert communication with full-duplex and half-duplex AF relays in [12]. The authors determined the optimal transmit power at the relay to maximize the covert rate while satisfying the covertness constraint. In [13], the authors considered beamformer design for decode-and-forward (DF) relay-assisted covert communication. In the presence of both direct and relay links, they provided an algorithm to jointly design the beamformers at the transmitter and the relay. A covert communication system in which relays and friendly jammers coexist was introduced in [14]. A relay selection strategy and power control were proposed, along with corresponding cooperative jamming. In these works, the focus was mainly on passive wardens who attempt to detect the covert transmission without taking active actions.

In recent works, the active warden, who poses a greater threat, has been considered in covert communication systems [15,16,17,18,19,20,21,22]. Contrary to the passive warden, who only attempts to detect the covert transmission, the active warden can not only detect the covert transmission but also transmit jamming signals to disrupt the reception of the covert message at the receivers [15]. In [16], the authors investigated the performance of covert communication from the perspective of the active warden under channel uncertainty. The active warden was assumed to eavesdrop on the transmitter and simultaneously generate artificial noise toward the receiver in full-duplex mode. They showed that the active warden was able to inject artificial noise covertly with perfect CSI of the jamming link, regardless of CSI uncertainties at the transmitter and receiver. Depending on the availability of CSI at the transmitter, the performance of covert communication was analyzed in terms of the covertness outage probability [17]. To counter the threat posed by the active warden, efficient transmission strategies have been proposed for various covert communication systems, such as multi-channel communication [18], UAV-assisted communication [19], and short-packet communication [20]. For relay-assisted covert communications, transmission strategies against the active warden have been proposed, and their performance has been analyzed [21,22]. A power threshold game for covert communication with a single relay was presented in [21]. The authors proved that O(n) bits can be transmitted covertly in the proposed system, and the Nash equilibrium was obtained. In [22], the authors analyzed the performance of relay selection schemes in covert communication with multiple relays and an active warden. Specifically, they considered random relay selection and maximum channel relay selection schemes, which were originally developed for conventional relay systems without an active warden. The paper investigates the performance of these conventional schemes when applied to covert communication scenarios involving an active warden in terms of the detection error probability of the warden and the outage probability. The relay schemes in existing works were primarily designed for conventional communication systems, where the active warden is absent. Therefore, these schemes cannot efficiently counter the detection threats and jamming attacks posed by the active warden.

In this paper, we consider a two-phase DF relaying system, where the transmitter sends the covert message in the first phase, and the relay forwards it to the receiver in the second phase. From the active warden’s perspective, it is most effective to be located near the relay during both phases. In the first phase, the active warden can significantly disrupt covert message reception at the relay, and in the second phase, it can easily detect covert message transmission from the relay. In other words, to achieve covert communication with a high covert rate, it becomes critically threatening when the channel between the relay and the active warden is strong. Therefore, in covert communication with multiple relays, we consider a relay selection strategy to minimize the threat posed by the active warden. In our proposed relay selection scheme, we choose the relay with the lowest channel gain from the relay to the warden. For the proposed scheme, we analyze the theoretical performance and demonstrate performance improvements based on analytical results, specifically in terms of the warden’s detection error probability, the average received signal-to-interference plus noise ratio (SINR), and the outage probability. We then formulate a power allocation problem at the transmitter and the relay to maximize the covert rate. The numerical results validate our theoretical analysis, and we evaluate the performance of the proposed scheme in comparison to reference schemes.

The rest of this paper is organized as follows. In Section 2, we present the system configuration, signal model, and the proposed relay selection scheme. In Section 3, we analyze the theoretical performance, including the detection probability of the warden, the average received SINR, and the outage probability for the proposed scheme. We also formulate the power allocation problem to maximize the covert rate in this section. In Section 4, we validate our analytical analysis and evaluate the performance of the proposed scheme. Finally, the conclusion of this paper is provided in Section 5.

## 2. System Model

### 2.1. System Configuration

**Figure 1:**
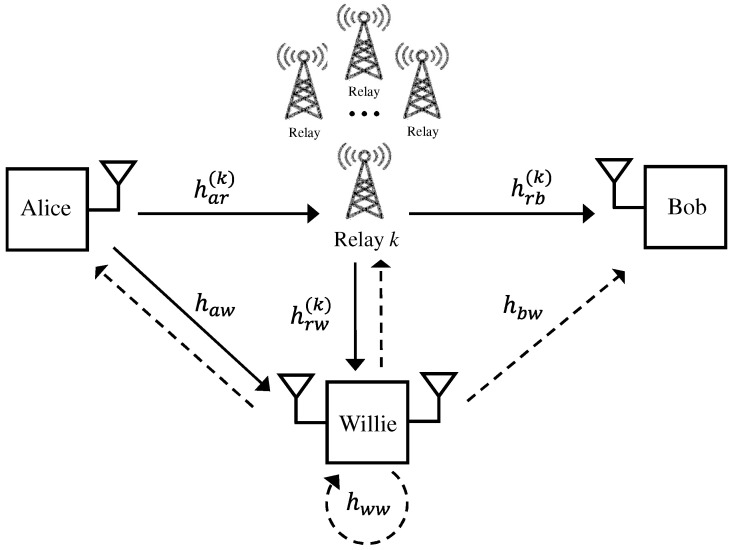
Covert communication with multiple relays and an active warden.

Our system model is illustrated in Figure 1. We consider a covert communication system with multiple relays, consisting of Alice (the transmitter), Bob (the receiver), Willie (the Warden), and *K* relays. In our system, Alice aims to covertly deliver the message to Bob. However, due to weak channel conditions between Alice and Bob, which may result from long distances or the presence of obstacles, Alice cannot transmit the covert message directly. Therefore, we assume that there is no direct channel between Alice and Bob for transmitting the covert message. To facilitate the delivery, the relay node assists in transmitting the covert message from Alice to Bob. For the relaying protocol, we consider a two-phase half-duplex DF relaying system, as it allows the relay to fully decode and re-encode the message, offering better control over the transmission process and facilitating covert constraints. Compared to AF relaying, which amplifies both signal and noise, DF reduces the risk of detection. While compress-and-forward (CF) is another alternative, it typically introduces greater complexity and latency, which may not be suitable for covert applications.

In the first phase, Alice may transmit the covert message to the relays with a certain probability or choose not to transmit. Among the *K* relays that receive the message from Alice, one relay is selected based on the relay selection method. In the second phase, the selected relay forwards the covert message to Bob. During both phases, Willie observes Alice and the relays, attempting to detect the covert message transmission. As mentioned in the previous section, we consider an active warden, who not only attempts to detect covert transmissions as a traditional warden but also actively sends jamming signals to disrupt covert message reception at the relays and at Bob. Thus, during both phases, the relays and Bob receive jamming signals from Willie and attempt to decode the covert message while treating the jamming as interference. Since Willie operates in full-duplex mode—simultaneously receiving covert signals and transmitting jamming signals—he experiences self-interference through the self-interference channel.

In our system model, we assume that every node has a single antenna. The channels from Alice to the *k*-th relay and Willie are denoted by $h_{ar}^{\left(k\right)}$ for k∈{1,…,K} and $h_{aw}$, respectively. The channels from the *k*-th relay to Willie and Bob are defined by $h_{rw}^{\left(k\right)}$ and $h_{rb}^{\left(k\right)}$, respectively. We assume that the system operates in time division duplex (TDD), where uplink and downlink channels use the same frequency band. In TDD, the channel from node *i* to node *j* is equal to the channel from node *j* to node *i* due to the channel reciprocity, i.e., $h_{ij}=h_{ji}$. Thus, the channels from Willie to the *k*-th relay and Bob are given by $h_{rw}^{\left(k\right)}$ and $h_{bw}$, respectively. The self-interference channel at Willie is denoted by $h_{ww}$. We consider a rich scattering environment, where there is no line-of-sight propagation between nodes. Therefore, every channel is modeled by an independent Rayleigh fading channel such that $h_{ij}∼\mathrm{CN}\left(0,\gamma_{ij}\right)$ and $h_{ij}^{\left(k\right)}∼\mathrm{CN}\left(0,\gamma_{ij}^{\left(k\right)}\right)$ for i,j∈{a,b,r,w} (this model assumes a rich scattering environment, where the effects of multipath propagation are captured statistically through fading. Although inter-symbol interference is not explicitly modeled, this simplification is made for analytical tractability). The average channel gains of $h_{ij}$ and $h_{ij}^{\left(k\right)}$ are given by $\gamma_{ij}≜E{\left|h_{ij}\right|}^{2}$ and $\gamma_{ij}^{\left(k\right)}≜E|h_{ij}^{\left(k\right)}{|}^{2}$, respectively. In addition, we assume that all channels remain unchanged during two phases.

The entire process of covert communication in our system is divided into three main steps, which are described in detail below.

1. Step 1: Channel Estimation
  Each relay estimates the channel to Bob based on a pilot sequence transmitted by Bob. Then, in a time-division manner, each relay broadcasts its own pilot sequence to allow the other relays and Alice to estimate the reciprocal channels.
  Since Willie is assumed to be an untrusted user within the network (rather than an external observer), it also participates in the pilot exchange process. Therefore, the channel between each relay and Willie can be estimated as part of the standard CSI acquisition.
  The relevant CSI—including relay-to-Bob, inter-relay, and relay-to-Willie channels—is securely shared with Alice through a low-rate control channel or via pre-established coordination mechanisms.
  This assumption allows Alice to have access to the relay-to-Willie channel gains without requiring any external sensing or feedback from Willie, as the channel is naturally observed during the standard CSI exchange.
2. Step 2: Relay Selection
  Based on the received CSI, Alice applies a relay selection strategy—such as selecting the relay with the weakest link to Willie—and determines the optimal relay for covert forwarding. The selection result is then communicated to Bob using secure signaling, which is either pre-agreed or implemented through an implicit coordination scheme (e.g., timer-based contention or scheduled slot allocation).
  To maintain covertness, all coordination for relay selection is assumed to occur via secure or implicit means, such as low-rate signaling prior to transmission, pre-agreed scheduling, or timer-based contention protocols. No overt signaling is assumed during the data transmission phase, and we assume that Willie cannot observe the selection process directly.
3. Step 3: Covert Transmission
  Once the relay (say, Relay $k^{*}$) is selected, Alice initiates covert communication through the selected relay, which forwards the message to Bob. During this step, the adversary (Willie) attempts to detect the presence of covert transmission. An active jammer may simultaneously transmit interference to improve covertness. The proposed model assumes that Willie cannot observe the relay selection signaling or infer coordination from the communication structure.

### 2.2. Signal Model

In the first phase, Alice decides to transmit or not transmit the covert message to relays with a certain probability. These correspond to two hypotheses, $H_{0}$ (do not transmit the covert message) and $H_{1}$ (transmit the covert message). Thus, in the first phase, the transmit signal at Alice can be represented by (in this work, we consider the covert message to be transmitted over a fixed number of channel uses within a single communication interval. Although the message is modeled as a scalar signal in the analytical derivations for simplicity, it represents a block of symbols transmitted over these channel uses. The total transmission duration is assumed to be sufficiently short so that channel conditions remain quasi-static during the communication interval).

(1) $$\begin{array}{c} x\left[1\right]=\left\{\begin{array}{cc} 0, & H_{0}\\ \sqrt{P_{a}}x_{C}, & H_{1} \end{array}\right., \end{array}$$

where $x_{C}\in C$ is the covert message with $E|x_{C}{|}^{2}=1$, and $P_{a}$ is the transmit power for the covert message at Alice with a maximum power constraint of $P_{a}\leq P_{a}^{\max}$.

As mentioned in the previous subsection, Willie sends the jamming signal to disrupt the reception of the relays and Bob during both phases. Therefore, the received signal at the *k*-th relay is given by

(2) $$\begin{array}{c} y_{r}^{\left(k\right)}\left[1\right]=\left\{\begin{array}{cc} \sqrt{P_{w}}h_{rw}^{\left(k\right)}x_{J}+n_{r}^{\left(k\right)}, & H_{0}\\ \sqrt{P_{a}}h_{ar}^{\left(k\right)}x_{C}+\sqrt{P_{w}}h_{rw}^{\left(k\right)}x_{J}+n_{r}^{\left(k\right)} & H_{1} \end{array}\right., \end{array}$$

where $x_{J}$ is the jamming signal with $E|x_{J}{|}^{2}=1$, and $P_{w}$ is the transmit power for the jamming signal at Willie. To effectively disrupt the transmission, Willie must have knowledge of the CSI between Alice, the relays, and Bob, as well as the transmit power of Alice and the relays, by optimizing the transmit power. In this paper, we assume that Willie does not have such information and thus uses a fixed transmit power $P_{w}$. Hence, Alice and the relays are aware of Willie’s fixed power. A circular symmetric complex Gaussian noise at the *k*-th relay is given by $n_{r}^{\left(k\right)}$, and we assume the noises at relays have the same variance $\sigma_{r}^{2}$, such that $n_{r}^{\left(k\right)}∼\mathrm{CN}\left(0,\sigma_{r}^{2}\right)$ for all *k*.

In the first phase, Willie listens to the transmission from Alice while sending the jamming signal, and thus the received signal at Willie is given by

(3) $$\begin{array}{c} y_{w}\left[1\right]=\left\{\begin{array}{cc} \sqrt{P_{w}}h_{ww}x_{J}+n_{w}, & H_{0}\\ \sqrt{P_{a}}h_{aw}x_{C}+\sqrt{P_{w}}h_{ww}x_{J}+n_{w}, & H_{1} \end{array}\right., \end{array}$$

where $n_{w}∼\mathrm{CN}\left(0,\sigma_{w}^{2}\right)$ is the complex Gaussian noise at Willie. In (3), we note that Willie experiences self-interference due to the jamming signal.

Among *K* relays that receive the signal from Alice, one relay is selected to forward the covert message to Bob. Let the $k^{★}$-th relay be the selected relay. Then, the $k^{★}$-th relay decodes the covert message and forwards it to Bob in the second phase. The transmit signal at the $k^{★}$-th relay can be represented by

(4) $$\begin{array}{c} x\left[2\right]=\left\{\begin{array}{cc} 0, & H_{0}\\ \sqrt{P_{r}}x_{C}, & H_{1} \end{array}\right., \end{array}$$

where $P_{r}$ is the transmit power for the covert message at the relay with a maximum power constraint of $P_{r}\leq P_{r}^{\max}$. In the second phase, since Willie also sends the jamming signal with a fixed power of $P_{w}$, the received signal at Bob is given by

(5) $$\begin{array}{c} y_{b}\left[2\right]=\left\{\begin{array}{cc} \sqrt{P_{w}}h_{bw}x_{J}+n_{b}, & H_{0}\\ \sqrt{P_{r}}h_{rb}^{\left(k^{★}\right)}x_{C}+\sqrt{P_{w}}h_{bw}x_{J}+n_{b}, & H_{1} \end{array}\right., \end{array}$$

where $n_{b}∼\mathrm{CN}\left(0,\sigma_{b}^{2}\right)$ is the complex Gaussian noise at Bob.

In the second phase, similar to (3), Willie experiences self-interference, and the received signal at Willie is represented by

(6) $$\begin{array}{c} y_{w}\left[2\right]=\left\{\begin{array}{cc} \sqrt{P_{w}}h_{ww}x_{J}+n_{w}, & H_{0}\\ \sqrt{P_{r}}h_{rw}^{\left(k^{★}\right)}x_{C}+\sqrt{P_{w}}h_{ww}x_{J}+n_{w}, & H_{1} \end{array}\right.. \end{array}$$

### 2.3. Relay Selection

As mentioned in the previous section, the key factor that critically affects the performance of covert communication with the active warden is the channel status from the relay to Willie, since Willie can detect the covert transmission and send jamming signals through this channel. Therefore, we propose a relay selection scheme to minimize the impact of the active warden’s channel. In the proposed scheme, we select the relay with the minimum channel gain between the relay and Willie, as follows:

(7) $$\begin{array}{cc} k^{★}=\underset{k\in \{1,\ldots ,K\}}{\mathrm{argmin}\;}\; & |h_{rw}^{\left(k\right)}{|}^{2}. \end{array}$$

As the reference schemes, we consider the random relay selection and maximum relay channel selection schemes. In the random relay selection scheme, the relay is chosen randomly to forward the covert message. In the maximum relay channel selection scheme, the end-to-end communication quality is constrained by the weaker link between the two hops: the Alice-to-relay channel and the relay-to-Bob channel. Therefore, the relay is selected by maximizing the minimum of the channel gains of the two hops, as given by

(8) $$\begin{array}{cc} k^{\max}=\underset{k\in \{1,\ldots ,K\}}{\mathrm{argmax}\;} & \min\left\{|h_{ar}^{\left(k\right)}{|}^{2},\;|h_{rb}^{\left(k\right)}{|}^{2}\right\}. \end{array}$$

The maximum relay channel selection scheme is the optimal relay selection strategy for the conventional DF relaying system, where Willie is not present, but it does not take the channels to Willie into account.

## 3. Performance Analysis of the Proposed Relay Selection Scheme

In this section, we analyze the performance of the proposed relay selection scheme based on minimizing the relay-to-Willie channel gain.

### 3.1. Detection Error Probability at Willie

Based on the Neyman–Pearson criterion, Willie decides between the hypotheses using the received power measured by a radiometer, as follows [7]:

(9) $$\begin{array}{c} Y_{w}\left[i\right]\underset{D_{0}}{\overset{D_{1}}{≷}}\phi ,\;i\in \left\{1,2\right\}, \end{array}$$

where $D_{0}$ and $D_{1}$ denote the decisions that Willie approves for hypotheses $H_{0}$ and $H_{1}$, respectively, and ϕ is a decision threshold. From (3) and (6), the received powers at Willie in the first and second phases are given, respectively, by [7]

(10) $$\begin{array}{cc} Y_{w}\left[1\right] & =\left\{\begin{array}{cc} P_{w}|h_{ww}{|}^{2}+\sigma_{w}^{2}, & H_{0}\\ P_{a}|h_{aw}{|}^{2}+P_{w}|h_{ww}{|}^{2}+\sigma_{w}^{2}, & H_{1} \end{array}\right., \end{array}$$

(11) $$\begin{array}{cc} Y_{w}\left[2\right] & =\left\{\begin{array}{cc} P_{w}|h_{ww}{|}^{2}+\sigma_{w}^{2}, & H_{0}\\ P_{r}|h_{rw}^{\left(k^{★}\right)}{|}^{2}+P_{w}|h_{ww}{|}^{2}+\sigma_{w}^{2}, & H_{1} \end{array}\right.. \end{array}$$

Meanwhile, Willie faces two types of detection errors: (1) miss detection, where Willie fails to detect the covert message transmission, and (2) false alarm, where Willie mistakenly identifies the transmission of a covert message when none is actually sent. Therefore, the detection error probability at Willie is given by the sum of the probabilities for the miss detection and false alarm as [22]

(12) $$\begin{array}{cc} \xi [i] & =P_{\mathrm{FA}}\left[i\right]+P_{\mathrm{MD}}\left[i\right],\;i\in \left\{1,2\right\}, \end{array}$$

where $P_{\mathrm{FA}}=\mathrm{Pr}\left(D_{1}|H_{0}\right)$ and $P_{\mathrm{MD}}=\mathrm{Pr}\left(D_{0}|H_{1}\right)$ are the false alarm and miss detection probabilities, respectively.

Since the detection error probabilities for Willie in both phases can be derived in the same manner, we first derive the detection error in the second phase, which is determined by the proposed relay selection. We assume that all channels follow the Rayleigh distribution, and thus, we obtain the false alarm probability in the second phase as follows:

(13) $$\begin{array}{cc} P_{\mathrm{FA}}\left[2\right] & =\mathrm{Pr}\left(P_{w}|h_{ww}{|}^{2}+\sigma_{w}^{2}\geq \phi \right) \end{array}$$

(14) $$\begin{array}{cc} \; & =\left\{\begin{array}{cc} \exp\left(-\frac{\phi -\sigma_{w}^{2}}{P_{w}\gamma_{ww}}\right), & \phi \geq \sigma_{w}^{2}\\ 1, & \mathrm{otherwise} \end{array}.\right. \end{array}$$

From (14), we can observe that the false alarm probability is determined by Willie’s jamming strategy, which includes the transmit power of the jamming signal and the self-interference channel gain.

Since we select the $k^{★}$-th relay with the minimum channel gain from the relay to Willie, Willie’s miss detection probability in the second phase is obtained by

(15) $$\begin{array}{cc} \; & \;\;P_{\mathrm{MD}}\left[2\right]=\mathrm{Pr}\left\{P_{r}|h_{rw}^{\left(k^{★}\right)}{|}^{2}+P_{w}|h_{ww}{|}^{2}+\sigma_{w}^{2}\leq \phi \right\} \end{array}$$

(16) $$\begin{array}{cc} \; & =\;\left\{\begin{array}{cc} \;\;1-\frac{P_{r}\gamma_{rw}^{\min}}{P_{r}\gamma_{rw}^{\min}-P_{w}\gamma_{ww}}\exp\left(-\frac{\phi -\sigma_{w}^{2}}{P_{r}\gamma_{rw}^{\min}}\right)\\ \;\;\;\;\;\;\;+\frac{P_{w}\gamma_{ww}}{P_{r}\gamma_{rw}^{\min}-P_{w}\gamma_{ww}}\exp\left(-\frac{\phi -\sigma_{w}^{2}}{P_{w}\gamma_{ww}}\right),\; & \;\;\;\;\;\phi \geq \sigma_{w}^{2}\\ \;\;0, & \;\;\;\;\;\mathrm{otherwise}, \end{array}\right. \end{array}$$

where, based on the minimum distribution of the independent exponential random variables, $\gamma_{rw}^{\min}$ is obtained as

(17) $$\begin{array}{c} \gamma_{rw}^{\min}={\left(\frac{1}{\gamma_{rw}^{\left(1\right)}}+\frac{1}{\gamma_{rw}^{\left(2\right)}}\ldots +\frac{1}{\gamma_{rw}^{\left(K\right)}}\right)}^{-1}. \end{array}$$

From (16), we can observe that the miss detection probability is a decreasing function of the average channel gain from the relay to Willie. Therefore, the proposed relay selection scheme can effectively increase Willie’s miss detection probability by reducing the average channel gain as shown in (17).

The detection error probability of Willie in the second phase is obtained by summing (14) and (16) as follows:

(18) $$\begin{array}{c} \;\xi \left[2\right]\;=\;\left\{\begin{array}{cc} \;\;\;1\;-\;\left(\frac{P_{r}\gamma_{rw}^{\min}}{P_{r}\gamma_{rw}^{\min}-P_{w}\gamma_{ww}}\right)\\ \;\;\;\times \;\left\{\exp\left(-\frac{\phi -\sigma_{w}^{2}}{P_{r}\gamma_{rw}^{\min}}\right)\;-\;\exp\left(-\frac{\phi -\sigma_{w}^{2}}{P_{w}\gamma_{ww}}\right)\right\}, & \;\;\;\phi \geq \sigma_{w}^{2},\\ \;\;\;1, & \;\;\;\mathrm{otherwise}. \end{array}\right. \end{array}$$

For the case where $\phi \geq \sigma_{w}^{2}$, from (18), the first derivative of the detection error with respect to ϕ is obtained as follows:

(19) $$\begin{array}{cc} \frac{\partial \xi [2]}{\partial \phi } & =\left(\frac{P_{r}\gamma_{rw}^{\min}}{P_{r}\gamma_{rw}^{\min}-P_{w}\gamma_{ww}}\right)\{\frac{1}{P_{r}\gamma_{rw}^{\min}}\exp\left(-\frac{\phi -\sigma_{w}^{2}}{P_{r}\gamma_{rw}^{\min}}\right)\\ \; & \;-\frac{1}{P_{w}\gamma_{ww}}\exp\left(-\frac{\phi -\sigma_{w}^{2}}{P_{w}\gamma_{ww}}\right)\}, \end{array}$$

and we can obtain $\phi^{★}$ that satisfies ∂ξ[2]/∂ϕ=0 as

(20) $$\begin{array}{c} \phi^{★}=\left(\frac{P_{r}P_{w}\gamma_{rw}^{\min}\gamma_{ww}}{P_{r}\gamma_{rw}^{\min}-P_{w}\gamma_{ww}}\right)\ln\left(\frac{P_{r}\gamma_{rw}^{\min}}{P_{w}\gamma_{ww}}\right)+\sigma_{w}^{2}. \end{array}$$

Let f(ϕ) represent (19) as a function of ϕ. Then, we have f(ϕ)<0 for $\phi <\phi^{★}$ and f(ϕ)>0 for $\phi >\phi^{★}$. Therefore, the detection error probability ξ[2] is a quasi-convex function of the decision threshold ϕ when $\phi \geq \sigma_{w}^{2}$. Consequently, the optimal decision threshold that minimizes the detection error probability can be obtained by (20).

Substituting (20) into (18), the minimized detection error probability of Willie in the second phase is obtained by

(21) $$\begin{array}{cc} \xi^{★}\left[2\right] & =\left\{\begin{array}{cc} 1-{\left(\frac{P_{r}\gamma_{rw}^{\min}}{P_{w}\gamma_{ww}}\right)}^{-\left(\frac{P_{w}\gamma_{ww}}{P_{r}\gamma_{rw}^{\min}-P_{w}\gamma_{ww}}\right)},\; & \phi \geq \sigma_{w}^{2},\\ 1, & \mathrm{otherwise}. \end{array}\right. \end{array}$$

In the same manner as in obtaining (21), the minimized detection error probability of Willie in the first phase is obtained as follows:

(22) $$\begin{array}{cc} \xi^{★}\left[1\right] & =\left\{\begin{array}{cc} 1-{\left(\frac{P_{a}\gamma_{aw}^{\left(k^{★}\right)}}{P_{w}\gamma_{ww}}\right)}^{-\left(\frac{P_{w}\gamma_{ww}}{P_{a}\gamma_{aw}^{\left(k^{★}\right)}-P_{w}\gamma_{ww}}\right)}, & \phi \geq \sigma_{w}^{2},\\ 1, & \mathrm{otherwise}. \end{array}\right. \end{array}$$

The detection error probabilities presented in (21) and (22) represent the minimum values achievable by Willie under the assumed jamming and system strategy considered in this paper, provided that he has perfect knowledge of all channel gains and the exact transmit powers $P_{r}$ and $P_{a}$. Due to the quasi-convex nature of the detection error probability with respect to the decision threshold, Willie can minimize it by optimizing the threshold accordingly. However, in practice, Willie does not have access to such perfect information, and thus cannot attain this minimum detection error probability. From the perspective of Alice and the covert communication system, these values are used as conservative benchmarks when designing transmission strategies under worst-case assumptions.

In order to compare the detection error probabilities for the proposed and reference schemes in the second phase, we consider the case where the channel gains of the relays are equal, i.e., $\gamma_{rw}^{\left(1\right)}=\ldots =\gamma_{rw}^{\left(K\right)}=\gamma_{rw}$. In this case, the minimum average channel gain from the relay to Willie in (17) is simplified as $\gamma_{rw}^{\min}=\gamma_{rw}/K$ (even under heterogeneous channel conditions, the proposed scheme can still provide performance gains by statistically reducing the strength of the relay-to-warden channels—equivalently, the jamming interference received from the warden—as shown in (17). However, to clearly and intuitively observe the performance improvement of the proposed scheme, we adopted the idealized assumption of identical average channel gains for analytical tractability). The random relay selection and maximum relay channel selection schemes do not take into account the channel between the relay and Willie when selecting the relay. Thus, for $\phi \geq \sigma_{w}^{2}$, the detection error probability for these schemes, $\xi^{\mathrm{con}}\left[2\right]$, is obtained by replacing $\gamma_{rw}^{\min}$ with $\gamma_{rw}$ in (21) as follows:

(23) $$\begin{array}{cc} \xi^{\mathrm{con}}\left[2\right] & =1-{\left(\frac{P_{r}\gamma_{rw}}{P_{w}\gamma_{ww}}\right)}^{-\left(\frac{P_{w}\gamma_{ww}}{P_{r}\gamma_{rw}-P_{w}\gamma_{ww}}\right)}. \end{array}$$

The proposed scheme reduces Willie’s detection probability, 1−ξ, compared to the reference schemes, as follows:

(24) $$\begin{array}{c} 1-\xi^{★}\left[2\right]=\alpha \left(1-\xi^{\mathrm{con}}\left[2\right]\right), \end{array}$$

where α is derived by substituting (21) and (23) into (24):

(25) $$\begin{array}{cc} \alpha = & {\left(\frac{1}{K}\right)}^{-\left(\frac{KP_{w}\gamma_{ww}}{P_{r}\gamma_{rw}-KP_{w}\gamma_{ww}}\right)}\\ \; & \times {\left(\frac{P_{r}\gamma_{rw}}{P_{w}\gamma_{ww}}\right)}^{-\left\{\frac{\left(K-1\right)P_{r}P_{w}\gamma_{rw}\gamma_{ww}}{(P_{r}\gamma_{rw}-KP_{w}\gamma_{ww})\left(P_{r}\gamma_{rw}-P_{w}\gamma_{ww}\right)}\right\}}. \end{array}$$

Also, in (25), we note that α is less than one for K>1 and is the decreasing function of *K*.

To more explicitly observe the performance of the proposed scheme, we consider the special case where $P_{r}\gamma_{rw}=P_{w}\gamma_{ww}$. In this case, the detection probability for the reference schemes becomes $1-\xi^{\mathrm{con}}\left[2\right]=1$, and α is simplified to $\alpha ={\left(\frac{1}{K}\right)}^{-\left(\frac{K}{1-K}\right)}$. Therefore, in this case, the detection error probability for the reference schemes becomes $\xi^{\mathrm{con}}\left[2\right]=0$. However, for the proposed scheme, the detection error probability at Willie is given by

(26) $$\begin{array}{c} \xi^{★}\left[2\right]=1-{\left(\frac{1}{K}\right)}^{-\left(\frac{K}{1-K}\right)}. \end{array}$$

From (26), we can observe that the proposed scheme increases the detection error probability of Willie as *K* increases. Furthermore, the effect of the proposed scheme is significant even for a small number of *K*.

### 3.2. Received SINR and Outage Probability

From the received signals at the $k^{★}$-th relay and Bob given in (3) and (5), respectively, the received SINR at the $k^{★}$-th relay in the first phase is obtained by

(27) $$\begin{array}{c} {\mathrm{SINR}}_{r}^{\left(k^{★}\right)}=\frac{P_{a}|h_{ar}^{\left(k^{★}\right)}{|}^{2}}{P_{w}|h_{rw}^{\left(k^{★}\right)}{|}^{2}+\sigma_{r}^{2}}, \end{array}$$

and the received SINR at Bob in the second phase is given by

(28) $$\begin{array}{c} {\mathrm{SINR}}_{b}=\frac{P_{r}|h_{rb}^{\left(k^{★}\right)}{|}^{2}}{P_{w}|h_{bw}{|}^{2}+\sigma_{b}^{2}}. \end{array}$$

In a high signal-to-noise ratio (SNR) region where $P_{a}\gg \sigma_{r}^{2}$ and $P_{w}\gg \sigma_{r}^{2}$, we compare the asymptotic behavior of the SINR at the relay for the proposed and reference schemes. For simplicity, we assume the case of equal average channel gains, where $\gamma_{ar}^{\left(1\right)}=\cdots =\gamma_{ar}^{\left(K\right)}=\gamma_{ar}$ and $\gamma_{rw}^{\left(1\right)}=\cdots =\gamma_{rw}^{\left(K\right)}=\gamma_{rw}$.

For the random relay selection scheme, we obtain the average received SINR in the high-SNR regime as

(29) $$\begin{array}{cc} {\bar{\mathrm{SINR}}}_{r}^{\mathrm{ran}} & ≜E\left[\frac{P_{a}|h_{ar}{|}^{2}}{P_{w}|h_{rw}{|}^{2}+\sigma_{r}^{2}}\right] \end{array}$$

(30) $$\begin{array}{cc} \; & =\frac{P_{a}\gamma_{ar}}{P_{w}\gamma_{rw}+\sigma_{r}^{2}} \end{array}$$

(31) $$\begin{array}{cc} \; & \approx \frac{P_{a}\gamma_{ar}}{P_{w}\gamma_{rw}}. \end{array}$$

In the maximum relay channel selection scheme, for the comparison, we assume that the relay is chosen based on the Alice-to-the relay channel and $|h_{ar}^{\max}{|}^{2}≜\max_{k=1,\ldots ,K}{\left|h_{ar}^{\left(k\right)}\right|}^{2}$. The average channel gain of the Alice-to-selected relay link is given by $E[|h_{ar}^{\max}{|}^{2}]=H_{K}\gamma_{ar}$, where $H_{K}≜\sum_{k=1}^{K}\frac{1}{k}$ denotes the *K*-th harmonic number. Then, the average received SINR in the high-SNR regime is obtained by

(32) $$\begin{array}{cc} {\bar{\mathrm{SINR}}}_{r}^{\max} & ≜E\left[\frac{P_{a}|h_{ar}^{\max}{|}^{2}}{P_{w}|h_{rw}{|}^{2}+\sigma_{r}^{2}}\right] \end{array}$$

(33) $$\begin{array}{cc} \; & =\frac{P_{a}H_{K}\gamma_{ar}}{P_{w}\gamma_{rw}+\sigma_{r}^{2}}, \end{array}$$

(34) $$\begin{array}{cc} \; & \approx H_{K}\frac{P_{a}\gamma_{ar}}{P_{w}\gamma_{rw}} \end{array}$$

(35) $$\begin{array}{cc} \; & =H_{K}\cdot {\bar{\mathrm{SINR}}}_{r}^{\mathrm{ran}}. \end{array}$$

For the proposed relay selection scheme, the average channel gain of the selected relay-to-warden link is given by $\gamma_{rw}^{\min}=\gamma_{rw}/K$. Accordingly, in the high-SNR regime, the average received SINR can be expressed as

(36) $$\begin{array}{cc} {\bar{\mathrm{SINR}}}_{r}^{\left(k^{★}\right)} & ≜E\left[\frac{P_{a}|h_{ar}^{\left(k^{★}\right)}{|}^{2}}{P_{w}|h_{rw}^{\left(k^{★}\right)}{|}^{2}+\sigma_{r}^{2}}\right] \end{array}$$

(37) $$\begin{array}{cc} \; & =\frac{P_{a}\gamma_{ar}}{\frac{1}{K}P_{w}\gamma_{rw}+\sigma_{r}^{2}} \end{array}$$

(38) $$\begin{array}{cc} \; & \approx K\frac{P_{a}\gamma_{ar}}{P_{w}\gamma_{rw}} \end{array}$$

(39) $$\begin{array}{cc} \; & =K\cdot {\bar{\mathrm{SINR}}}_{r}^{\mathrm{ran}}. \end{array}$$

From (35) and (39), we can observe that both the maximum relay channel selection and the proposed selection schemes increase the received SINR of the random selection proportionally to the number of the relays, i.e., *K*. For K>1, the proposed relay selection scheme increases the SINR linearly with *K*, while the maximum relay channel selection scheme increases the SINR with $H_{K}\left(<K\right)$.

For a given target SINR Γ, we derive the outage probability, which is the probability that the received SINR is less than Γ. Since the channel gain in Rayleigh fading follows an exponential distribution, by using the cumulative distribution function (CDF) of the exponential distribution, we obtain the outage probability in the first phase as follows:

(40) $$\begin{array}{cc} p_{\mathrm{out}}^{\left[1\right]} & =\mathrm{Pr}\left({\mathrm{SINR}}_{r}^{\left(k^{★}\right)}<\Gamma \right)\\ \; & =\mathrm{Pr}\left(\frac{P_{a}|h_{ar}^{\left(k^{★}\right)}{|}^{2}}{P_{w}|h_{rw}^{\left(k^{★}\right)}{|}^{2}+\sigma_{c}^{2}}<\Gamma \right)\\ \; & =1-E\left[\exp\left(-\frac{\Gamma \left(P_{w}|h_{rw}^{\left(k^{★}\right)}{|}^{2}+\sigma_{r}^{2}\right)}{P_{a}\gamma_{ar}^{\left(k^{★}\right)}}\right)\right]\\ \; & =1-\exp\left(-\frac{\Gamma \sigma_{r}^{2}}{P_{a}\gamma_{ar}^{\left(k^{★}\right)}}\right)E\left[\exp\left(-\frac{\Gamma P_{w}|h_{rw}^{\left(k^{★}\right)}{|}^{2}}{P_{a}\gamma_{ar}^{\left(k^{★}\right)}}\right)\right]\\ \; & =1-\exp\left(-\frac{\Gamma \sigma_{r}^{2}}{P_{a}\gamma_{ar}^{\left(k^{★}\right)}}\right)\left(\frac{P_{a}\gamma_{ar}^{\left(k^{★}\right)}}{\Gamma P_{w}\gamma_{rw}^{\min}+P_{a}\gamma_{ar}^{\left(k^{★}\right)}}\right). \end{array}$$

Similarly, the outage probability in the second phase is obtained as follows:

(41) $$\begin{array}{cc} p_{\mathrm{out}}^{\left[2\right]} & =\mathrm{Pr}\left({\mathrm{SINR}}_{b}<\Gamma \right)\\ \; & =1-\exp\left(-\frac{\Gamma \sigma_{b}^{2}}{P_{r}\gamma_{rb}^{\left(k^{★}\right)}}\right)\left(\frac{P_{r}\gamma_{rb}^{\left(k^{★}\right)}}{\Gamma P_{w}\gamma_{bw}+P_{r}\gamma_{rb}^{\left(k^{★}\right)}}\right). \end{array}$$

In the two-phase DF relaying system, the outage event occurs when it takes place in at least one phase. In other words, the outage event occurs unless no outage event occurs in both phases. Therefore, based on (40) and (41), the outage probability for the proposed relay selection is obtained by

(42) $$\begin{array}{cc} p_{\mathrm{out}} & =1-\left(1-p_{\mathrm{out}}^{\left[1\right]}\right)\left(1-p_{\mathrm{out}}^{\left[2\right]}\right)\\ \; & =1-\exp\left\{-\Gamma \left(\frac{\sigma_{c}^{2}}{P_{a}\gamma_{ar}^{\left(k^{★}\right)}}+\frac{\sigma_{b}^{2}}{P_{r}\gamma_{rb}^{\left(k^{★}\right)}}\right)\right\}\\ \; & \;\;\;\;\;\times \left(\frac{P_{a}\gamma_{ar}^{\left(k^{★}\right)}}{\Gamma P_{w}\gamma_{rw}^{\min}+P_{a}\gamma_{ar}^{\left(k^{★}\right)}}\right)\;\left(\frac{P_{r}\gamma_{rb}^{\left(k^{★}\right)}}{\Gamma P_{w}\gamma_{bw}+P_{r}\gamma_{rb}^{\left(k^{★}\right)}}\right). \end{array}$$

### 3.3. Power Allocation for Covert Rate Maximization

In the first phase, from (27), the achievable rate for the covert message at the $k^{★}$-th relay is obtained by

(43) $$\begin{array}{cc} R_{C}^{\left[1\right]} & =\log_{2}\left(1+{\mathrm{SINR}}_{r}^{\left(k^{★}\right)}\right)\\ \; & =\log_{2}\left(1+\frac{P_{a}|h_{ar}^{\left(k^{★}\right)}{|}^{2}}{P_{w}|h_{rw}^{\left(k^{★}\right)}{|}^{2}+\sigma_{r}^{2}}\right). \end{array}$$

From (39), we note that the proposed scheme asymptotically improves the achievable rate in (43) as the number of relays *K* increases. Based on (28), the achievable rate for the covert message at Bob in the second phase is obtained by

(44) $$\begin{array}{cc} R_{C}^{\left[2\right]} & =\log_{2}\left(1+{\mathrm{SINR}}_{b}\right)\\ \; & =\log_{2}\left(1+\frac{P_{r}|h_{rb}^{\left(k^{★}\right)}{|}^{2}}{P_{w}|h_{bw}{|}^{2}+\sigma_{b}^{2}}\right). \end{array}$$

In the covert communication with DF relaying, the covert message must be successfully decoded at both the relay and Bob. Therefore, the achievable covert rate is limited by the minimum of the achievable rates at the relay and Bob [23]. Taking the outage probability into account, the covert rate is defined as follows:

(45) $$\begin{array}{c} R_{C}=\left(1-p_{\mathrm{out}}\right)\min\left\{R_{C}^{\left[1\right]},\;R_{C}^{\left[2\right]}\right\}. \end{array}$$

To achieve the covertness of the communication during both phases, it is guaranteed that the detection error probability of Willie must be larger than predefined requirement of the system, i.e., ξ[i]≥1−ϵ,0≤ϵ≤1,i∈{1,2}, where ϵ is the covertness constraint. Therefore, the power allocation problem at Alice and the relay, aimed at maximizing the covert rate while satisfying the covertness constraint, is formulated as follows:  

(46) $$\begin{array}{cc} \left(P1\right)\;\;\underset{P_{a},P_{r}}{\mathrm{maximize}\;} & R_{C} \end{array}$$

(47) $$\begin{array}{cc} \mathrm{subject}\;\mathrm{to} & \xi^{★}\left[1\right]\geq 1-\epsilon ,\;\xi^{★}\left[2\right]\geq 1-\epsilon \end{array}$$

(48) $$\begin{array}{cc} \; & P_{a}\leq P_{a}^{\max},\;P_{r}\leq P_{r}^{\max}, \end{array}$$

where $\xi^{★}\left[1\right]$ and $\xi^{★}\left[2\right]$ are given in (22) and (21), respectively. The transmit power at Alice, i.e., $P_{a}$, and at the relay, i.e., $P_{r}$, is coupled in $p_{\mathrm{out}}$ in (42), but $p_{\mathrm{out}}$ is a monotonic decreasing function with respect to both $P_{a}$ and $P_{r}$. For fixed jamming power $P_{w}$, $R_{C}^{\left[1\right]}$ and $R_{C}^{\left[2\right]}$ are increasing functions of $P_{a}$ and $P_{r}$, respectively. Since the covertness constraints $\xi^{★}\left[1\right]$ and $\xi^{★}\left[2\right]$ are the functions of $P_{a}$ and $P_{r}$, the problem (P1) can be decoupled into two sub-problems that maximize $P_{a}$ and $P_{r}$ while satisfying the covertness constraints $\xi^{★}\left[1\right]$ and $\xi^{★}\left[2\right]$, respectively. The sub-problem for the power allocation at Alice is formulated by

(49) $$\begin{array}{cc} \left(P1.1\right)\mathrm{maximize} & P_{a} \end{array}$$

(50) $$\begin{array}{cc} \mathrm{subject}\;\mathrm{to} & \xi^{★}\left[1\right]\geq 1-\epsilon , \end{array}$$

(51) $$\begin{array}{cc} \; & P_{a}\leq P_{a}^{\max}. \end{array}$$

Since $\xi^{★}\left[1\right]$ in (22) is a decreasing function of $P_{a}$, the solution of the problem (P1.1) is given by

(52) $$\begin{array}{c} P_{a}^{★}=\min\left\{{\bar{P}}_{a},\;P_{a}^{\max}\right\}, \end{array}$$

where ${\bar{P}}_{a}$ is the power that satisfies $\xi^{★}\left[1\right]=1-\epsilon$, and it can be easily obtained using a one-dimensional search algorithm, such as the bisection method. A detailed algorithm is given in Algorithm 1.
**Algorithm 1** Bisection Algorithm to Find Optimal Transmit Power at Alice $P_{a}$1:**Input:** Maximum transmit power $P_{\max}$, target covertness level ϵ, tolerance δ, Willie’s detection error probability in first phase $\xi^{★}\left[1\right]\left(P_{a}\right)$2:**Output:** Power value $P_{a}^{★}$ such that $|\xi^{★}\left[1\right]\left(P_{a}^{★}\right)-\left(1-\epsilon \right)|<\delta$3:**if** $\xi^{★}\left[1\right]\left(P_{a}^{\max}\right)>1-\epsilon$ **then**4:    **Return** $P_{a}^{\max}$5:**end if**6:$P_{\mathrm{low}}\leftarrow 0$, $P_{\mathrm{high}}\leftarrow P_{a}^{\max}$7:**repeat**8:      $P_{\mathrm{mid}}\leftarrow \frac{P_{\mathrm{low}}+P_{\mathrm{high}}}{2}$9:      **if** $\xi^{★}\left[1\right]\left(P_{\mathrm{mid}}\right)>1-\epsilon$ **then**10:        $P_{\mathrm{low}}\leftarrow P_{\mathrm{mid}}$11:    **else**12:        $P_{\mathrm{high}}\leftarrow P_{\mathrm{mid}}$13:    **end if**14:**until** $|\xi^{★}\left[1\right]\left(P_{\mathrm{mid}}\right)-\left(1-\epsilon \right)|<\delta$15:**Return** $P_{\mathrm{mid}}$

The sub-problem for the power allocation at the relay is also formulated by

(53) $$\begin{array}{cc} \left(P1.2\right)\mathrm{maximize} & P_{r} \end{array}$$

(54) $$\begin{array}{cc} \mathrm{subject}\;\mathrm{to} & \xi^{★}\left[2\right]\geq 1-\epsilon \end{array}$$

(55) $$\begin{array}{cc} \; & P_{r}\leq P_{r}^{\max}, \end{array}$$

and the solution of the problem (P1.2) is given by

(56) $$\begin{array}{c} P_{r}^{★}=\min\left\{{\bar{P}}_{r},\;P_{r}^{\max}\right\}, \end{array}$$

where ${\bar{P}}_{r}$ is the power that satisfies $\xi^{★}\left[2\right]=1-\epsilon$.

By using $P_{a}^{★}$ and $P_{r}^{★}$, the covert rate for the proposed relay selection scheme is represented as follows:

(57) $$\begin{array}{c} R_{C}\left(P_{a}^{★},P_{r}^{★}\right)\;=\;\left(1\;-\;p_{\mathrm{out}}\left(P_{a}^{★},P_{r}^{★}\right)\right)\;\min\;\left\{R_{C}^{\left[1\right]}\left(P_{a}^{★}\right),R_{C}^{\left[2\right]}\left(P_{r}^{★}\right)\;\right\}. \end{array}$$

## 4. Numerical Results

In this section, we verify our analytical analysis via simulations and evaluate the performance of the proposed relay selection by comparing it with the reference schemes (since there are few existing relay selection schemes specifically designed for the environments with the active warden, which is the main focus of this study, we used the random relay selection and maximum relay channel selection schemes as references to clearly demonstrate the advantages of our proposed method that considers the channel between the relay and Willie). As mentioned in Section 2.3, for reference schemes, we consider the random relay selection and maximum relay channel selection [22]. In our simulations, we assume that the average channel gains from Alice to the relays are equal and set to $\gamma_{ar}^{\left(1\right)}=\cdots =\gamma_{ar}^{\left(K\right)}=\gamma_{ar}=1$. The average channel gains from the relays to Bob are also assumed to be equal to $\gamma_{rb}^{\left(1\right)}=\cdots =\gamma_{rb}^{\left(K\right)}=\gamma_{rb}=1$. The average channel gains of Alice-to-Willie and Willie-to-Bob are set to $\gamma_{aw}=\gamma_{bw}=0.5$, and the average channel gain of the self-interference at Willie is set to $\gamma_{ww}=0.5$. We consider equal average channel gains from the relays to Willie that aer set to $\gamma_{rw}^{\left(1\right)}=\cdots =\gamma_{rw}^{\left(K\right)}=\gamma_{rw}=1$, unless otherwise specified. The noise powers at all nodes are set equal to $\sigma_{r}^{2}=\sigma_{w}^{2}=\sigma_{b}^{2}=\sigma^{2}$. The maximum transmit power budgets of Alice and the relays are assumed to be equal, and thus their transmit SNR becomes $\rho ≜\frac{P_{a}^{\max}}{\sigma^{2}}=\frac{P_{r}^{\max}}{\sigma^{2}}$. The transmit SNR of Willie is defined by $\rho_{w}≜\frac{P_{w}}{\sigma^{2}}$. We also assume that the target SINR for the outage event is set to Γ=1, and the covertness constraint is set to ϵ=0.25.

**Figure 2:**
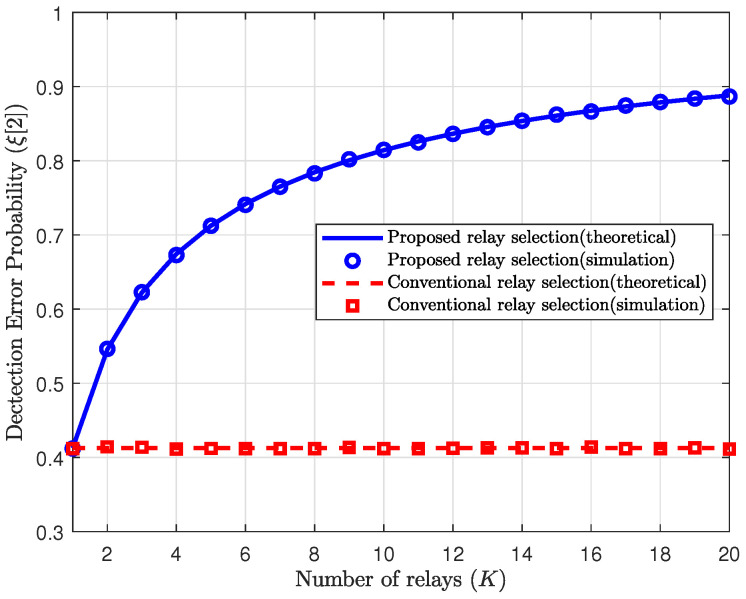
Detection error probability in the second phase vs. the number of relays with ρ=10dB and $\rho_{w}=5\;\mathrm{dB}$.

**Figure 3:**
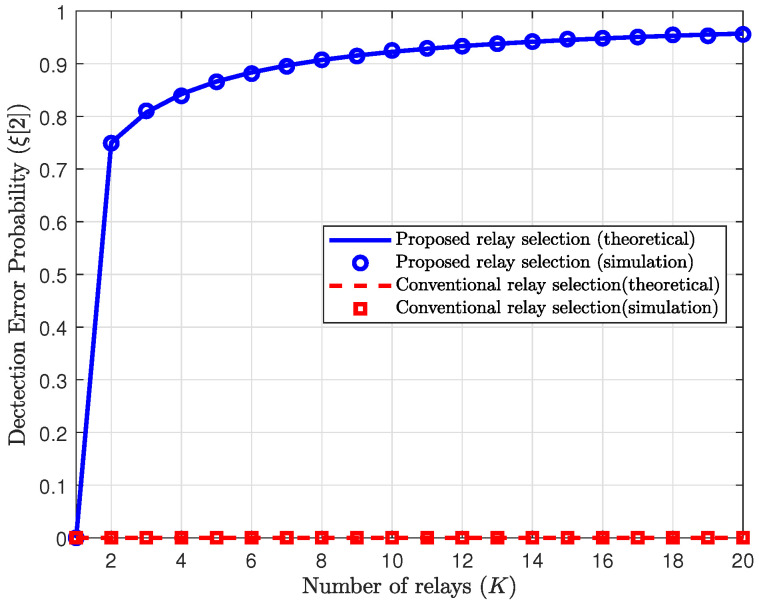
Detection error probability in the second phase vs. the number of relays with $\rho =\rho_{w}=10\;\mathrm{dB}$.

We first evaluate the performance of the proposed scheme in terms of the detection error probability of Willie in the second phase. In Figure 2 and Figure 3, the detection error probabilities of Willie in the second phase are plotted with respect to the number of the relay, i.e., *K*, for the proposed and reference schemes. The theoretical detection error probability for the proposed scheme is given in (21), and the detection error probability for the reference scheme is obtained by replacing $\gamma_{rw}^{\min}$ with $\gamma_{rw}$ in (21). To ensure the verification of the theoretical results, all channels are generated more than $10^{5}$ times for the simulation. In this figure, we assume that the relay uses full power with the maximum power budget $P_{r}=P_{r}^{\max}$.

In Figure 2, the transmit SNRs of the relay and Willie are set to ρ=10dB and $\rho_{w}=5\;\mathrm{dB}$, respectively. The conventional relay selection refers to both the random relay selection and the maximum channel relay selection, since Willie’s detection error probabilities for both schemes are identical. From Figure 2, we first observe that our theoretical results closely match with the simulations. As mentioned in Section 3.1, in the reference schemes, the relay is chosen regardless of the channels to Willie, and thus the detection error probability is not affected by *K*. However, the proposed scheme selects the relay with the minimum channel gain to Willie, thereby minimizing the signal leakage to Willie. Thus, the proposed scheme increases the detection error probability of Willie with *K* in the second phase, as shown in (24). Furthermore, we can check that the proposed scheme effectively increases the detection error probability of Willie even for a small or moderate number of relays, e.g., *K* such that K<10.

Figure 3 shows the detection error probabilities of Willie with $\rho =\rho_{w}=10\;\mathrm{dB}$, where $P_{r}\gamma_{rw}=P_{w}\gamma_{ww}$. As mentioned in Section 3.1, the detection error probabilities of the reference schemes become 0 in this special case. Therefore, in the reference schemes, the relay must significantly reduce its transmit power to satisfy the covertness constraint. In this case, for the proposed scheme, the detection error probability of Willie is given by (26), and it increases as *K* increases. From Figure 3, we can observe that the effect of the proposed scheme is significant even for a small number of relays, e.g., *K* such that K<3. Therefore, the relay in the proposed scheme can use significantly more power for covert message transmission while satisfying the covertness constraint, as compared to the reference schemes.

**Figure 4:**
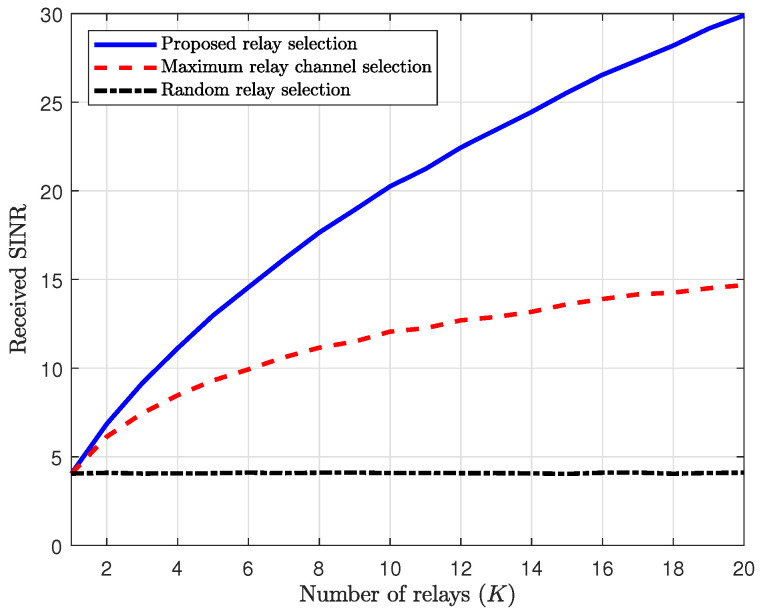
Received SINR of relay vs. the number of relays.

Figure 4 illustrates the received SINRs at the relay as a function of the number of relays, *K*. In this figure, since we aim to observe the asymptotic behavior of the received SINR at the relay in the high-SNR regime, we set the transmit SNRs of the relay and Willie to $\rho =\rho_{w}=20\;\mathrm{dB}$. Also, we assume that Alice uses full power with the maximum power budget $P_{a}=P_{a}^{\max}$. As shown in (31), we first observe that the random relay selection cannot achieve the diversity gain of *K* relays. In contrast, both the proposed and the maximum relay channel selection schemes can achieve the diversity gain of *K* relays, as shown in (39) and (35), respectively. The received SINR of the relay in the proposed scheme linearly increases according to the number of relays, while in the maximum relay channel selection scheme, it asymptotically increases with the *K*-th harmonic number, $H_{K}$ (where $H_{K}<K$).

**Figure 5:**
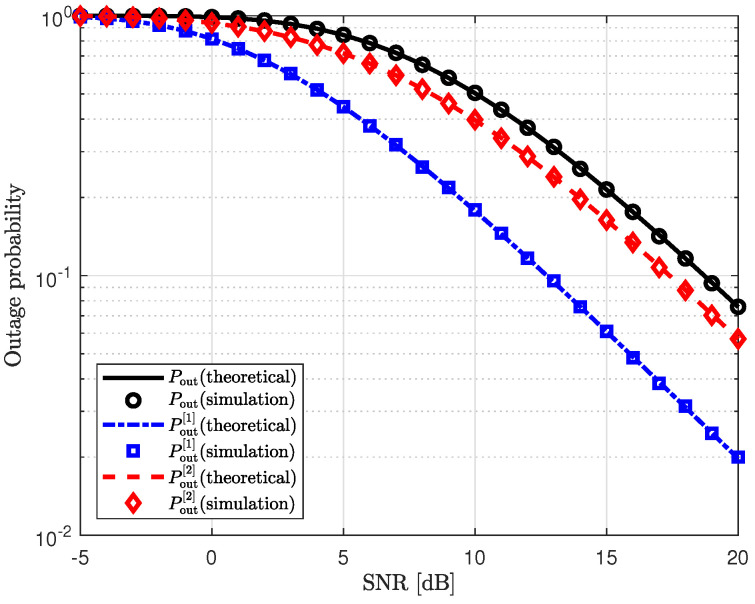
Outage probability vs. transmit SNR (ρ).

We verify our theoretical results for the outage probability through simulations in Figure 5. Figure 5 shows the outage probability of the proposed scheme as a function of the transmit SNR, along with the outage probabilities in the first and second phases. The theoretical outage probability of the proposed scheme is given in (42). The outage probabilities in the first and second phases are provided in (40) and (41), respectively. For the simulation results, the channels are generated over $10^{5}$ iterations. The transmit SNR of Willie is set to $\rho_{w}=5\;\mathrm{dB}$. In this figure, we can see that our theoretical results for the outage probability are closely aligned with the simulation results. Compared to the outage probability in the second phase, the proposed scheme significantly reduces the outage probability in the first phase by effectively mitigating the interference from Willie.

**Figure 6:**
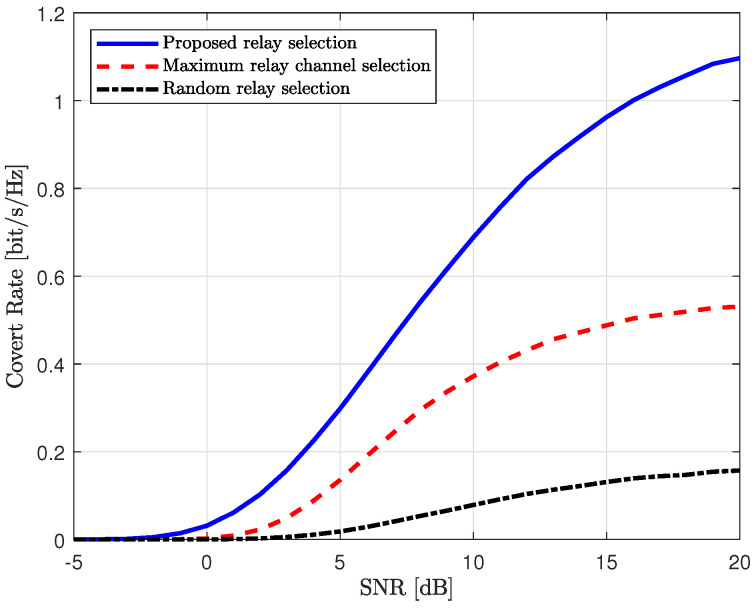
Covert rate vs. transmit SNR (ρ).

**Figure 7:**
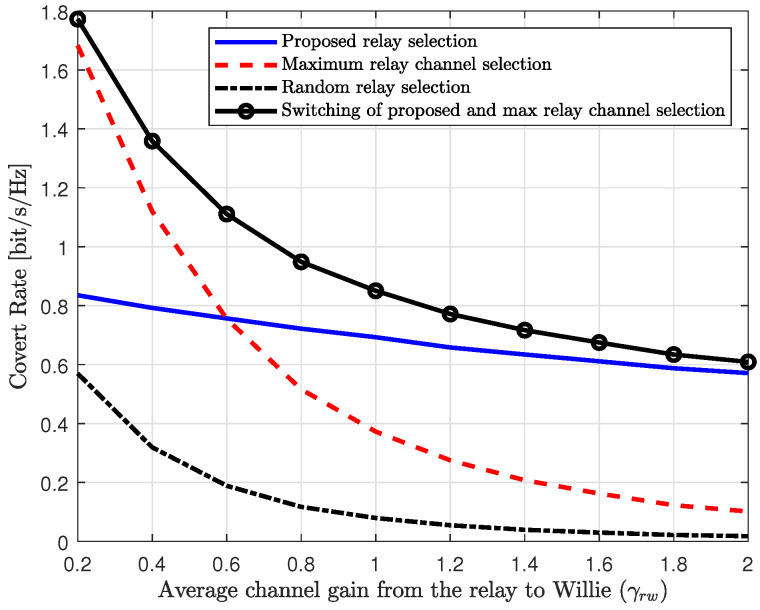
Covert rate vs. average channel gain from the relay to Willie ($\gamma_{rw}$).

We evaluate the performance of the proposed scheme with the reference schemes in terms of the covert rate in Figure 6 and Figure 7. The achievable covert rate of the proposed scheme is defined in (57), where the transmit powers at Alice and the relay are optimized to maximize the covert rate while satisfying the covertness constraint. For reference schemes, the transmit powers at Alice and the relay are also optimized to maximize their cover rates. The transmit SNRs at Alice, the relay, and Willie are set equal to $\rho =\rho_{w}$.

In Figure 6, the covert rates of the proposed scheme and the reference schemes are plotted in accordance with the transmit SNR. As mentioned previously, the average channel gain from the relay to Willie and the covertness constraint are set to $\gamma_{rw}=1$ and ϵ=0.25, respectively. In this figure, the proposed scheme outperforms the reference schemes, with the performance gap increasing in the high-SNR regime. For the configuration with a relatively strong channel from Willie, the performance of the reference schemes can degrade significantly due to the strong interference from Willie. Additionally, as shown in Figure 2 and Figure 3, the relay must reduce the transmit power for the covert message in order to satisfy the covertness constraint. However, the proposed scheme effectively reduces interference from Willie by selecting the relay with minimum channel gain from Willie. Furthermore, the proposed scheme allows the relay to use more power to forward the covert message while satisfying the covertness constraint, as shown in Figure 2 and Figure 3.

**Figure 8:**
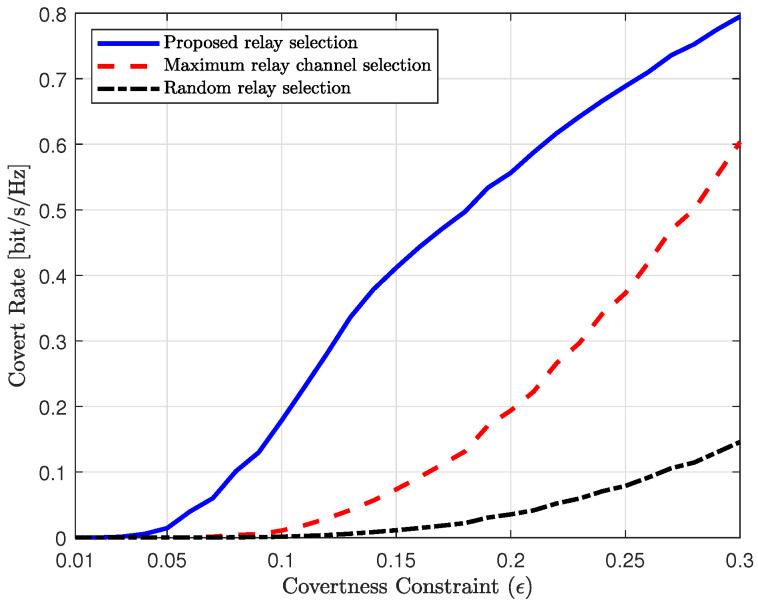
Covert rate vs. covertness constraint (ϵ).

Figure 8 shows the covert rate of the proposed and reference schemes as a function of the covertness constraint. The transmit SNR is set to 10 dB, i.e., ρ=10dB. It can be observed that the covert rate increases for all schemes as the covertness constraint becomes more relaxed. However, the proposed scheme and the maximum relay channel selection scheme exhibit a steeper increase in covert rate compared to the random relay selection scheme. In the regime of tight covertness constraints (e.g., ϵ<0.2), the performance of the conventional schemes is highly limited, whereas the proposed scheme achieves significantly higher covert rates. This is because the conventional schemes consider only the strength of the transmission channels, while the proposed scheme minimizes the signal leakage from the relay to Willie. As the covertness constraint becomes more relaxed, the performance of the maximum channel relay selection scheme approaches that of the proposed scheme, since under loose constraints, the channel strength to Bob becomes more dominant than minimizing the leakage to Willie. Consequently, the proposed scheme significantly outperforms the conventional schemes in scenarios requiring high covertness.

Figure 7 illustrates the effect of the average channel gain between the relay and Willie on the performance of the relay selection schemes. In Figure 7, the covert rates of the proposed and reference schemes are shown as a function of the average channel gain from the relay to Willie, $\gamma_{rw}$. In this figure, we can observe that the covert rates of the random relay selection and the maximum relay channel selection scheme decrease significantly as the average channel gain to Willie increases. Specifically, since the maximum relay channel selection scheme is optimal for the DF relaying without Willie, it outperforms the proposed scheme when the average channel gain to Willie is weak (e.g., $\gamma_{rw}<0.6$). However, it experiences significant performance degradation when Willie’s channel is strong (e.g., $\gamma_{rw}>1.4$). In contrast, the proposed scheme provides robust performance, with performance degradation due to the increase in Willie’s channel gain being marginal. Therefore, for the moderate or strong channels to Willie (e.g., $\gamma_{rw}>0.6$), the proposed scheme outperforms the reference schemes. To account for a general channel configuration, we plot a switching scheme between the proposed scheme and the maximum relay channel selection scheme. In the switching scheme, the relay selection scheme that provides the higher covert rate for a given channel configuration is chosen. In Figure 7, we can observe that the switching scheme provides higher performance compared to the other schemes by selecting the optimal strategy for the given channel configuration. However, the switching scheme requires the CSIs of all channels in the system and involves solving the power optimization for all relays to be implemented, which increases implementation complexity. Therefore, developing a simple and efficient algorithm that offers robust performance under general channel configurations remains an ongoing research topic.

## 5. Conclusions

In two-phase DF relay-assisted covert communication, we proposed the relay selection where Willie sends jamming signals to interfere with the covert transmission. In order to minimize the performance degradation caused by Willie, the relay that has the minimum channel gain to Willie is selected to forward the covert message. In the analytical results, we demonstrated that the proposed scheme effectively increases the detection error probability at Willie in the second phase, allowing the relay to use more power to forward the covert message while still satisfying the covertness constraint. We also showed that the received SINR of the relay increases asymptotically with the number of relays. By optimizing the transmit power at Alice and the relay, the proposed scheme can increase the covert rate, especially in scenarios with a strong channel to Willie. The numerical results confirmed that the analytical results align closely and showed that the proposed scheme can achieve the higher covert rate compared to reference schemes.

This work has considered a simplified fixed-power jammer model to facilitate initial analysis and design. However, in practical scenarios, jammers may adapt their power dynamically to disrupt communications more effectively. In future work, investigating relay selection schemes under more sophisticated and adaptive jamming strategies will be important to enhance system robustness and applicability in realistic environments. Furthermore, the current model treats the covert message as a scalar value transmitted within a single communication interval with fixed channel uses. In reality, covert messages consist of multiple symbols transmitted over varying durations, and the message length critically impacts detection performance. Incorporating explicit temporal dynamics and variable message lengths into the covert communication model will significantly increase analysis complexity and is therefore an important direction for future research. Additionally, this work assumes that the adversary Willie is an untrusted node within the network who participates in the pilot exchange process. This enables channel estimation and simplifies analysis; however, it may not capture more adversarial scenarios where Willie remains silent or avoids pilot transmission. Future research should address cases with incomplete or unavailable CSI regarding the adversary, potentially employing blind or indirect estimation techniques.

## Data Availability

Data are contained within the article.
